# The Timing of Pregnancies After Bariatric Surgery has No Impact on Children’s Health—a Nationwide Population-based Registry Analysis

**DOI:** 10.1007/s11695-022-06346-9

**Published:** 2022-11-07

**Authors:** Hannes Beiglböck, Eric Mörth, Berthold Reichardt, Tanja Stamm, Bianca Itariu, Jürgen Harreiter, Jakob Eichelter, Gerhard Prager, Alexandra Kautzky-Willer, Peter Wolf, Michael Krebs

**Affiliations:** 1 Division of Endocrinology and Metabolism, Department of Internal Medicine III, Medical University of Vienna, Währinger Gürtel 18-20, 1090 Vienna, Austria; 2 Department of Informatics, University of Bergen, 5008 Bergen, Norway; 3 Mohn Medical Imaging and Visualization Centre, Haukeland University Hospital, 5021 Bergen, Norway; 4 Austrian Social Health Insurance Fund, 7000 Eisenstadt, Austria; 5 Center for Medical Statistics, Informatics and Intelligent Systems, Institute for Outcomes Research, Medical University of Vienna, Währinger Gürtel 18-20, 1090 Vienna, Austria; 6 Ludwig Boltzmann Institute for Arthritis and Rehabilitation, Vienna, Austria; 7 Department of General Surgery, Division of Visceral Surgery, Medical University of Vienna, Währinger Gürtel 18-20, 1090 Vienna, Austria

**Keywords:** Bariatric surgery, Pregnancies, Infertility, Registry analysis, Healthcare research, Health outcomes

## Abstract

**Purpose:**

Bariatric surgery has a favorable effect on fertility in women. However, due to a lack of data regarding children’s outcomes, the ideal time for conception following bariatric surgery is unknown. Current guidelines advise avoiding pregnancy during the initial weight loss phase (12–24 months after surgery) as there may be potential risks to offspring. Thus, we aimed to analyze health outcomes in children born to mothers who had undergone bariatric surgery. The surgery-to-delivery interval was studied.

**Materials and Methods:**

A nationwide registry belonging to the Austrian health insurance funds and containing health-related data claims was searched. Data for all women who had bariatric surgery in Austria between 01/2010 and 12/2018 were analyzed. A total of 1057 women gave birth to 1369 children. The offspring’s data were analyzed for medical health claims based on International Classification of Diseases (ICD) codes and number of days hospitalized. Three different surgery-to-delivery intervals were assessed: 12, 18, and 24 months.

**Results:**

Overall, 421 deliveries (31%) were observed in the first 2 years after surgery. Of these, 70 births (5%) occurred within 12 months after surgery. The median time from surgery to delivery was 34 months. Overall, there were no differences noted in frequency of hospitalization and diagnoses leading to hospitalization in the first year of life, regardless of the surgery-to-delivery interval.

**Conclusion:**

Pregnancies in the first 24 months after bariatric surgery were common. Importantly, the surgery-to-delivery interval had no significant impact on the health outcome of the children.

**Graphical Abstract:**

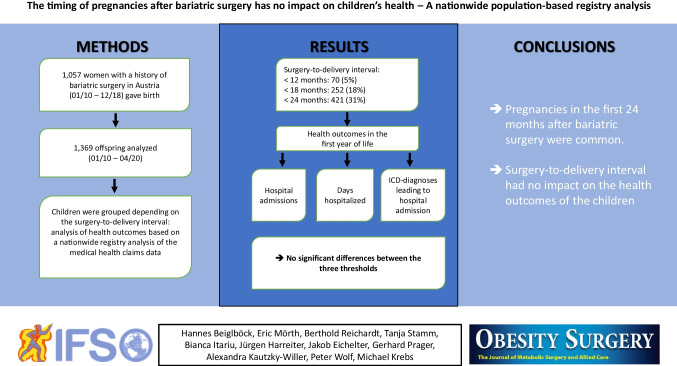

## Introduction

Obesity is a risk factor for infertility in women and weight loss after bariatric surgery has been shown to reverse this [1, 2]. Moreover, it was demonstrated that bariatric surgery had a favorable effect on sexual hormones in women with morbid obesity [3, 4]. Obesity is also a major risk factor for gestational diabetes (GDM), which itself is associated with higher health risks, not only for the women but also for their children [5]. Likewise, maternal obesity is a known risk factor for preterm delivery [6]. Some studies demonstrated that bariatric surgery reduced the likelihood of gestational diabetes but was associated with an increased risk of small for gestational age newborns [7–9]. Additionally, it was shown that children born to women who had bariatric surgery had a higher risk of perinatal complications compared to non-operated controls [10]. Bariatric surgery was also associated with an increased risk of preterm birth [11]. However, another study demonstrated that bariatric surgery reduced the risk of adverse delivery outcomes compared to women without bariatric surgery [12]. The time elapsed between bariatric surgery and birth could thus also substantially impact neonatal outcomes [10] and clinical practice guidelines recommend a period of 12–18 months after surgery during which conception should be avoided [13]. Other guidelines advise that pregnancy should be avoided in the weight loss phase following bariatric surgery [14, 15]. However, evidence from large, registry-type studies in a real-world setting is sparse. Therefore, this study aimed to analyze the impact of the timing of pregnancies after bariatric surgery on children’s health outcomes in a real-world, population-based registry study.

## Methods

### Design

Medical health claims data from the Austrian health insurance system which covers 98% of the total Austrian population were retrospectively analyzed. The data structure provided by the Austrian health insurance was previously described by Beiglböck et al. [16]. Data belonging to women who had undergone bariatric surgery between 01/2010 and 12/2018 and who gave birth after the surgery were extracted and analyzed. Data from their respective offspring was also analyzed. The children’s data comprised the period from 01/2010 to 04/2020. Three predefined surgery-to-delivery intervals were compared: 12 months, 18 months, and 24 months. We obtained the children’s date of birth, date of death (if applicable), number of hospitalizations, number of days hospitalized, and the coded diagnoses according to the International Statistical Classification of Diseases and Related Health Problems (ICD)-10 [17]. The used ICD-10 codes ranged from A00 to Z99 and are depicted in Table 2. Codes in a category, e.g., “A,” and “B” were aggregated, except for the ICD-category P00-P96 which is of special interest regarding health-related problems in newborns (see Table 3 for detailed information and description of each subgroup in category “P”). The mothers’ data included date of birth, date of death (if applicable), age at operation, age at death (if applicable), and type of bariatric surgical procedure based on medical single procedure (MEL)-codes [16]. The ethical committee of the Medical University of Vienna (No 2052/2018) approved the study protocol.

### Statistical Analysis

We used R (https://www.r-project.org/) for reshaping the data sets and Microsoft Excel (Microsoft, 2022) and SPSS (IBM, version 27) for the analysis. Data distribution was checked by visualization using histograms. We calculated means and standard deviations for normally distributed variables and alternatively, if this was not the case, medians and interquartile ranges (IQR). Furthermore, chi-squared tests were used to compare diagnoses between the reference points after bariatric surgery. Additionally, we compared the number of days hospitalized with Mann–Whitney *U* tests. Furthermore, unpaired *T*-tests were performed to compare the age of the mothers between the different groups (12 months, 18 months, 24 months). As this study was of an exploratory nature and was designed to generate hypotheses, corrections for multiple testing were not implemented. Because the lengths of observation periods differed, diagnoses and number of days in hospital were only calculated for the first year of the child’s life. The significance level for statistical analyses was set at *p* < 0.05.

## Results

Overall, 14,681 women who had undergone bariatric surgery between 01/2010 and 12/2018 were eligible for the analysis. Of these, 1057 women gave birth to 1369 children within the 01/2010 to 04/2020 observation period. Moreover, 271 women gave birth a second time, and 39 women delivered three times within the observation period. Two women gave birth to four children each, this was the maximum number observed. In addition, most of the children were male (736; 54%). The mean age of the mothers at the time of bariatric surgery was 27.1 ± 4.9 years, and the mean age at delivery was 30.4 ± 5.0 years. Generally, women who gave birth up to 24 months after bariatric surgery were older at the time of surgery compared to women who gave birth at least 24 months post-surgery (before: 28.1 ± 5.1 years, after: 26.7 ± 4.9 years; *p* = 0.000). Among all thresholds compared, women who gave birth before the specific reference point were older at the time of surgery compared to women who gave birth after (see Table 1). Moreover, comparing maternal age at delivery significant differences could be found for the 18 and 24 month thresholds (see Table 1).

**Table 1:** Demographics of mothers with a history of bariatric surgery and data on hospitalization of the offspring; data are given in median and interquartile range for normally distributed data and in mean and standard deviation for data not normally distributed

*Reference point after bariatric surgery*	*12 months*		*18 months*		*24 months*	
*Date of birth according to specific reference*	Before	After	Before	After	Before	After
*Births*	70 (5%)	1299 (95%)	252 (18%)	1117 (82%)	421 (31%)	948 (69%)
*Maternal age at operation*	28.7 ± 5.7*	27.0 ± 4.9*	28.0 ± 5.1*	26.9 ± 4.9*	28.1 ± 5.1*	26.7 ± 4.9*
*Maternal age at birth*	29.6 ± 5.7	30.5 ± 5.0	29.3 ± 5.1*	30.7 ± 5.0*	29.6 ± 5.1*	30.8 ± 4.9*
*Hospitalization in the first year of life in offspring*	26 (37%)	438 (34%)	79 (31%)	385 (35%)	136 (32%)	328 (35%)
*Days hospitalized in the first year of life in offspring*	7 [3;24]	6 [3;11]	6 [3;20]	6 [3;11]	6 [3;12]	6 [3;12]

^*^p < 0.05 regarding before/after for the specific reference point

Gastric bypass, performed in 72% of women, was the most frequent procedure, followed by sleeve gastrectomy which was performed in 17% of cases, and gastric banding which was performed in 10% of cases. Moreover, biliopancreatic diversion was performed in less than 1% (0.5%) of all cases. A total of 63 revision operations (5%) were recorded in the databases and in 4 cases (0.3%) a further operation following the revision operation was found.

The post-bariatric procedure deliveries were observed after a median of 34 months [interquartile range: 22–53 months]. A total of 70 births (5%) were recorded up to 12 months after bariatric surgery. Moreover, 182 deliveries (13%) occurred between 12 and 18 months post-surgery and 169 births (12%) were observed between 18 and 24 months after the bariatric procedure. Furthermore, most of the deliveries (948, 69%) in this analysis were recorded at least 24 months after the bariatric procedure.

Regarding the number of days spent in hospital in the first year of life, no significant differences were observed between the different reference points (see Table 1). In addition, the frequency of hospitalization in the first year of life was comparable between the specific reference points. However, regarding diagnoses in the first year of life, the diagnosis group H00-H59 (diseases of the eye and adnexa) demonstrated significant differences between the reference points. In contrast, no significant differences were observed for the most common diagnosis group P00–P96 (certain conditions originating in the perinatal period) (see Table 2). Furthermore, a sub analysis of this diagnosis group including the most important subgroup P05-P07, which comprises preterm birth and small for gestational age diagnoses, also showed no significant differences among the different thresholds (see Table 3).

**Table 2:** ICD-10 diagnoses [17] for conditions leading to the hospitalization of children born to mothers with a history of bariatric surgery. Hospitalizations occurred in the first year of life and are organized by surgery-to-delivery interval. n.a.: not applicable

*Date of birth after bariatric surgery*	*0–12 months*	*12–18 months*	*18–24 months*	*24 months or later*	*p-value*
*n*	**70**	**182**	**169**	**948**	
*ICD-codes*					
*A00–B99*	2 (3%)	2 (1%)	8 (5%)	29 (3%)	0.272
*C00–D48*	0	0	0	0	n.a
*D50–D89*	0	0	0	1 (0%)	0.987
*E00–E90*	0	1 (1%)	0	6 (1%)	0.964
*F00–F99*	0	0	0	3 (0%)	0.938
*G00–G99*	0	1 (1%)	0	5 (1%)	0.974
*H00–H59*	0	1 (1%)	4 (2%)	2 (0%)	0.005
*H60–H95*	1 (1%)	2 (1%)	0	2 (0%)	0.141
*I00–I99*	1 (1%)	0	1 (1%)	4 (0%)	0.645
*J00–J99*	5 (7%)	12 (7%)	14 (8%)	68 (7%)	0.947
*K00–K93*	3 (4%)	1 (1%)	5 (3%)	15 (2%)	0.131
*L00–L99*	1 (1%)	0	2 (1%)	4 (0%)	0.425
*M00–M99*	0	0	0	0	n.a
*N00–N99*	1 (1%)	1 (1%)	3 (2%)	16 (2%)	0.714
*O00–O99*	0	0	0	0	n.a
*P00–P96*	12 (17%)	24 (13%)	26 (15%)	160 (17%)	0.707
*Q00–Q99*	2 (3%)	10 (5%)	7 (4%)	40 (4%)	0.810
*R00–R99*	2 (3%)	3 (2%)	10 (6%)	38 (4%)	0.223
*S00–T98*	3 (4%)	4 (2%)	6 (4%)	20 (2%)	0.508
*V01–Y98*	0	0	0	0	n.a
*Z00–Z99*	0	1 (1%)	0	6 (1%)	0.964
*U00–U85*	0	0	0	0	n.a

A00-B99 certain infectious and parasitic diseases; C00-D48 neoplasms; D50-D89 diseases of the blood and blood-forming organs and certain disorders involving the immune mechanism; E00-E90 endocrine, nutritional and metabolic diseases; F00-F99 mental and behavioral disorders; G00-G99 diseases of the nervous system; H00-H59 diseases of the eye and adnexa; H60-H95 diseases of the ear and mastoid process; I00-I99 diseases of the circulatory system; J00-J99 diseases of the respiratory system; K00-K93 diseases of the digestive system; L00-L99 diseases of the skin and subcutaneous tissue; M00-M99 diseases of the musculoskeletal system and connective tissue; N00-N99 diseases of the genitourinary system; O00-O99 pregnancy, childbirth and the puerperium; P00-P96 certain conditions originating in the perinatal period; Q00-Q99 congenital malformations, deformations, and chromosomal abnormalities; R00-R99 symptoms, signs and abnormal clinical and laboratory findings, not elsewhere classified; S00-T98 injury, poisoning and certain other consequences of external causes; V01-Y98 external causes of morbidity and mortality; Z00-Z99 factors influencing health status and contact with health services and U00-U85 codes for special purposes

**Table 3:** ICD-10 diagnoses within the category P00 – P96 (certain conditions originating in the perinatal period) [17] leading to the hospitalization of children born to mothers with a history of bariatric surgery. Hospitalizations occurred in the first year of life and are organized by surgery-to-delivery interval. *n.a.*, not applicable

*Date of birth after bariatric surgery*	*0–12 months*	*12–18 months*	*18–24 months*	*24 months or later*	*p-value*
*n*	**70**	**182**	**169**	**948**	
*ICD-codes*					
*P00-P04*	0	0	1 (1%)	4	0.956
*P05–P08*	7 (10%)	13 (7%)	16 (9%)	84 (9%)	0.856
*P10–P15*	0	0	0	0	n.a
*P20–P29*	3 (4%)	6 (3%)	0	30 (3%)	0.809
*P35–P39*	1 (1%)	0	0	13 (1%)	0.743
*P50–P61*	1 (1%)	4 (2%)	0	13 (1%)	0.775
*P70–P74*	0	1 (1%)	0	6 (1%)	0.964
*P75–P78*	0	0	0	0	n.a
*P80–P83*	0	0	0	1 (0%)	0.987
*P90–P96*	0	0	0	9 (1%)	0.746

P00-P04 fetus and newborn affected by maternal factors and by complications of pregnancy, labor and delivery; P05-P08 disorders related to length of gestation and fetal growth; P10-P15 birth trauma; P20-P29 respiratory and cardiovascular disorders specific to the perinatal period; P35-P39 infections specific to the perinatal period; P50-P61 haemorrhagic and hematological disorders of fetus and newborn; P70-P74 transitory endocrine and metabolic disorders specific to fetus and newborn; P75-P78 digestive system disorders of fetus and newborn; P80-P83 conditions involving the integument and temperature regulation of fetus and newborn and P90-P96 other disorders originating in the perinatal period

Overall, two mothers died during the observation period, 8 months and 21 months after delivery and 14 months and 39 months after bariatric surgery respectively. Additionally, a child whose mother delivered 39 months after bariatric surgery died 14 months after birth due to severe comorbidities including brain malformation and hypopituitarism.

## Discussion

This study shows that (i) early pregnancies after bariatric surgery are frequently observed but (ii) no significant impact of the surgery-to-delivery interval on the health outcomes of the offspring could be identified in the first year of life.

Bariatric surgery is linked to higher chances of pregnancy in patients with obesity and is associated with better delivery outcomes [12, 18]. However, due to the rising prevalence of obesity, and thus the rising number of bariatric surgeries, the management and timing of pregnancies after these procedures are of major interest [19].

The percentage of women who delivered early in this study (5%) is higher compared to other analyses reporting 2.4% and 4.3% within 12 months post-surgery respectively [12, 20]. With regards to the surgery-to-conception interval, other studies demonstrated that up to 36.8% of pregnancies following bariatric surgery occurred within 12 months of the surgery [21–23]. The differences in numbers of early pregnancies after surgery might be explained by the different study designs and the sample size available for the respective analyses. Moreover, our analysis found that approximately every third delivery (31%) after bariatric surgery was recorded within the first 24 months following surgery. This percentage is within the previously reported range of 18.4 to 44.9% [12, 20]. However, insufficient perioperative counseling regarding contraception might be a reason for the relatively high number of women conceiving before the recommended surgery-to-conception interval [24]. On the other hand, the mothers’ age at delivery in our study was comparable with those in other studies [10, 25, 26]. Additionally, the different surgical bariatric procedures used were comparable to Heusschen et al. [21]. This is important for comparison since a study showed that pregnancy outcomes after a bariatric procedure might vary among the different bariatric procedures [27]. In short, the most important finding of this study was that the surgery-to-delivery interval had no impact on the health outcomes for the offspring in the first year of life. This finding is in line with those showing that conception within 18 months following bariatric surgery had no significant impact on neonatal outcomes [28]. A further study also demonstrated that even in early conceptions within 12 months after bariatric surgery, perinatal outcomes were not worse compared to conceptions more than 12 months post-surgery [25]. Additionally, other analyses suggest that the time elapsed between sleeve gastrectomy and pregnancy had no impact on neonatal outcomes [26, 29]. However, it was shown that gestational weight gain was lower in pregnancies up to 12 months after surgery compared to pregnancies after 12 months post-surgery [23]. That might be of importance since it was shown that normal gestational weight gain was linked to favorable obstetric outcomes [30]. However, our study in a large registry analysis with more than 1300 mother–child pairs supports the findings from Rasteiro et al. They used a small cohort to demonstrate that no definitive threshold for postponing pregnancies after bariatric surgery could be identified [22]. When compared between the different threshold groups, a higher number of diseases in the diagnosis group H00-H059 (diseases of the eye and adnexa) was only observed in children born between 18 and 24 months post-bariatric surgery. These numbers may not be clinically relevant as they were generally low and most diseases diagnosed in the group were mild in nature. Conjunctivitis is one example of a condition featuring in this group. The most relevant diagnosis group is P00-P96 (certain conditions originating in the perinatal period) which comprises all diagnoses of well-known potential problems including being small for gestational age (ICD-code: P05) and preterm birth (ICD-code: P07). This group demonstrated no difference between the defined surgery-to-delivery intervals (see Table 2). The in-depth analysis of more specific subgroups of diagnoses revealed no differences between the groups (see Table 3). Additionally, no difference between the surgery-to-delivery interval and number of diagnoses in the ICD-group Q00-Q99 (congenital malformations, deformations and chromosomal abnormalities) was found. This is in line with others showing that even early pregnancies are not associated with higher rates of congenital malformations [25].

It is worth noting that the study has some important limitations due to the available data recorded in the national health funds databases. Furthermore, the retrospective study design is a major limitation. Some major parameters including body mass index, weight loss after surgery, weight gain during the pregnancy, nutritional status of mothers, comorbidities of mothers during pregnancy, and data on abortion were missing. The assessment of children’s health in our analysis was limited to data on hospitalization and diagnoses made in hospital. Thus, minor health issues in children not requiring hospitalization were not covered in this study. However, the specific structure of the health care system in Austria promotes hospital admissions and therefore, we are confident that the available data provides sufficient sensitivity to draw conclusions on clinically significant differences depending on the surgery-to-delivery intervals. The study also demonstrates major strengths. One example is the sample size of approximately 1300 mother–child-pairs with no selection bias due to the nature of the Austrian healthcare system. The focus on the first year of life is a further strength as most other analyses focus on the perinatal period. As the Austrian health care system covers the costs for bariatric surgery, almost all bariatric surgeries within the included period were recorded in the database and analyzed in this study.

In summary, our analysis demonstrated that approximately every third birth following bariatric surgery was observed up to 24 months post-surgery. Very early pregnancies up to 12 months post-surgery were also detected in our study; according to the latest guidelines these should be avoided [31]. However, based on this registry analysis, surgery-to-birth interval had no relevant impact on the assessed health outcomes of the children. Nevertheless, further studies on long-term outcomes for children of mothers who have had bariatric surgery are needed before clinical guidelines can be adapted.
